# Effects of exogenous α-oxoglutarate on proline accumulation, ammonium assimilation and photosynthesis of soybean seedling (*Glycine max(L.) Merr.*) exposed to cold stress

**DOI:** 10.1038/s41598-020-74094-w

**Published:** 2020-10-12

**Authors:** Zhijia Gai, Lei Liu, Jingtao Zhang, Jingqi Liu, Lijun Cai

**Affiliations:** 1 Heilongjiang Academy of Agricultural Sciences Postdoctoral Programme, Xuefu Road 368, Harbin City, 150086 Heilongjiang Province China; 2 Jiamusi Branch, Heilongjiang Academy of Agricultural Sciences, Anqing Street 531, Dongfeng District, Jiamusi City, 154007 Heilongjiang Province China; 3 College of Agronomy, Northeast Agricultural University, Changjiang Road 600, Harbin City, 150038 Heilongjiang Province China

**Keywords:** Plant sciences, Plant physiology, Plant stress responses

## Abstract

The objective of this study was to examine the effects of exogenous α-oxoglutarate on leaf proline accumulation, ammonium assimilation and photosynthesis of soybean when exposed to cold stress. To achieve this objective, exogenous α-oxoglutarate was sprayed to potted seedlings of Henong60 and Heinong48 at 0, 2.5, 5.0 and 7.5 mmol/L, identified as A_0_, A_2.5_, A_5.0_, and A_7.5_, respectively. Leaf samples were collected after cold stress of 24 h (S1 stage) and 48 h (S2 stage). The results indicated that exogenous α-oxoglutarate significantly enhanced leaf GS activity, NADP-GDH activity, glutamate content, proline content and photosynthesis of soybean seedling exposed to cold stress at S1 and S2 stages. The ammonium content in leaf was significantly decreased by exogenous α-oxoglutarate at both stages. 5.0 mmol/L of exogenous α-oxoglutarate is the optimum concentration in this study. Leaf proline content for Henong60 and Heinong48 at A_5.0_ was 37.53% and 17.96% higher than that at A_0_ at S1 stage, respectively. Proline content for Henong60 and Heinong48 increased by 28.82% and 12.41% at A_5.0_ and A_0_, respectively, at S2 stage. Those results suggested that exogenous α-oxoglutarate could alleviate the adverse effects of cold stress.

## Introduction

Low temperature often occurs during soybean seedling stage in Northeast China, which will pose great damage to soybean growth and development. Cold stress severely limits plant growth and leads to substantial productivity loss. Several plants species have evolved different mechanisms to minimize the adverse impact of cold stress^1^. Osmoregulation is an important mechanism that plants use to acclimate to different abiotic stresses. Proline is proposed to act as a main osmolyte in enhancing plant stress tolerance^2^.

In plants, proline accumulation has been reported to occur after cold stress^3^. Among the beneficial compounds, proline is greatly important for plants to cope with cold stress; the correlation between the acquisition of stress tolerance and proline accumulation has been verified^4^. In fact, proline is eventually synthesized from Glu in glutamate (Glu) pathway and ornithine (Orn) pathway^5^. Glutamate plays a significant role in amino acid metabolism in plants^6^. Glu biosynthesis is greatly linked with ammonium assimilation^6^. A mass of protein in plant can decompose into ammonium during osmotic stress^2^. Excessive ammonium can cause toxic effects on plants and must be rapidly transformed into non-toxic organic nitrogen compounds^7^. It has been demonstrated that GS/GOGAT cycle is the major route of ammonium assimilation in plants. Alternatively, NADH-dependent glutamate dehydrogenase (NADH-GDH; EC 1.4.1.2) might also catalyze ammonium incorporation into glutamate^7, 8^. The function of the alternative GDH pathway is still obscure and is believed to play a complementary role under conditions of environmental stress or excessive ammonia supply^9–11^.

2-oxoglutarate (2-OG), a key organic acid of the tricarboxylic acid (TCA) cycle^12, 13^ participates in a range of reactions in distinct plant cell compartments^14^, also being a key metabolite at the crossroads of carbon/nitrogen metabolism as it is required for ammonia assimilation^15, 16^. Some information is available on the effects of exogenous α-oxoglutarate on nitrogen assimilation in plants, such as rice^17^, tobacco^18, 19^, wheat^20^, and tomato^21^. Magalhaes et al.^21^ reported that NH_4_^+^ concentrations in tomato roots and shoots with exogenous α-oxoglutarate sharply decreased compared to control and the increase of NH_4_^+^ assimilation due to exogenous α-oxoglutarate clearly indicated that the availability of carbon skeletons is a key limiting factor for NH_4_^+^ assimilation in tomato plants. Yuan et al.^17^ investigated the role of α-oxoglutarate in regulation of carbon and nitrogen metabolisms in non-photosynthetic tissues of rice and found that exogenous α-oxoglutarate enhanced the activity of GOGAT (EC 1.4.1.14) and GS (EC 6.3.1.2) enzymes. And the increase in ammonium uptake and glutamate (Glu) was also observed in their study^17^. However, no information is available on the impacts of exogenous α-oxoglutarate on soybean seedling leaf ammonium assimilation and proline accumulation.

The objective of this investigation was to examine the impacts of exogenous α-oxoglutarate on ammonium assimilation proline accumulation and photosynthesis of soybean seedling exposed to cold stress (6 °C) in pot experiment. Through this study, we expected to understand the optimum dose of exogenous α-oxoglutarate applied to soybean seedling when exposed to cold stress. Our results could provide theoretical reference for taking measures against chilling in soybean production in Northeast China.

## Materials and methods

### Plant materials and growth conditions

A pot experiment was conducted at Experimental Station of Jiamusi Branch of Heilongjiang Academy of Agricultural Sciences, China. Experimental pot diameter is 14 cm and the depth is 16 cm. Each pot was filled with 500 g of soil. The soil used in the present study is the typical black soil with silty clay loam texture. Two spring soybean varieties Henong60 and Heinong48 were used in this experiment. Henong60, released in 2009, is cold-tolerant variety with 100-seed weight 18 g and plant height is 60 cm. The content of its protein and oil is 38.47% and 22.25%, respectively. The growth habit is determinate with white flowers. The effective accumulative temperature for maturity (≥ 10 °C) is about 2290 °C. Heinong48, released in 2004, is cold-sensitive variety with 100-seed weight 22 g and plant height 90 cm. The content of its protein and oil is 45.23% and 18.43%, respectively. The growth habit is semi-determinate with purple flowers. The effective accumulative temperature for maturity (≥ 10 °C) is about 2380 °C.

### Experimental design and sampling

The simulation of cold stress was conducted according to the method by Tambussi et al.^21^ with minor modifications. Soybean was not inoculated before seeding. Ten seeds of each cultivar were sown by hand in each pot. There are four soybean seedlings left in the pot after emergence. The seedlings of the two soybean varieties were grown in pots in field. Upon the appearance of the first true fully expanded leaves (V1 stage), 4 ml of exogenous α-oxoglutarate was sprayed to each pot of Henong60 and Heinong48. There were four exogenous α-oxoglutarate treatments including 0, 2.5, 5.0 and 7.5 mmol/L, identified as A_0_, A_2.5_, A_5.0_ and A_7.5_. Treated seedlings were transferred into controlled environment growth chambers at 14-h-light/10-h-dark. The light intensity was 120 µmol m^−2^ s^−1^ and the relative humidity was 65%. The cold stress degree is 6 °C. There were three replicates for each treatment and there were four pots for each replicates. Leaf samples for each treatment were collected after cold stress of 24 h (S1 stage) and 48 h (S2 stage).

### Determination of nitrogen metabolism

The activities of NADH-GDH and GS were determined according to Lu et al.^22^. One unit of NADH-GDH activity is defined as the reduction of 1 µmol of coenzyme (NADH) per min at 30 °C. One unit of GS activity is the amount of enzyme that catalyzes the formation of 1 µmol γ-glutamylhydroxamate per min at 37 °C.

### Determination of photosynthetic index

Photosynthetic rate was measured according to Gai et al.^23^. Photosynthetic rate was determined by using the CI-340 portable photosynthesis measuring system (CID, Inc., USA).

Measurement of chlorophyll content (*SPAD*) was conducted by using *SPAD*-502 Chlorophyll Meter Model (Konica Minolta Inc., Japan).

### Determination of proline

Free proline was measured according to the method by Bates et al.^24^ with some modifications. Proline was extracted from 1.0 g fresh leaves with 10 ml 3% sulphosalicylic acid at 100 °C for 10 min. 4 ml of the extract was then mixed with 4 ml ninhydrin reagent containing glacial acetic acid and incubated at 100 °C for 30 min. The reaction mixture was quickly cooled with running tap water. The colored reaction product was extracted with 8 ml toluene, and the absorbance of the toluene phase was measured at 520 nm.

### Determination of ammonium content

The ammonium content of leaves was determined following HPLC (Agilent 1100, USA) according to the method by Lu et al.^22^.

### Statistical analyses

All the data were statistically analyzed in SPSS 22 software. Analysis of variance (ANOVA) was used to test for significance, and significant differences (*P* < 0.05) between treatments were determined by using LSD test.

## Results and discussion

### Response of soybean fresh weight to exogenous α-oxoglutarate

The growth of soybean seedlings was monitored by measuring the fresh weight of soybean. The data in Table 1 showed that exogenous α-oxoglutarate has no significant effects on fresh weight for two soybean varieties at S1 and S2 stages. However, there was a significant difference in fresh weight between two soybean varieties at both stages. The fresh weight for Heinong48 was significantly greater than Henong60 at 0.001, 0.01, and 0.05 level at S1 and S2 stages, respectively. From S1 stage to S2 stage, the fresh weight for cold-resistant variety Henong60 and cold-sensitive variety Heinong48 decreased by 6.45% and 15.96%, respectively. There was lower reduction in fresh weight for Henong60 than Heinong48 when exposed to cold stress. Exogenous α-oxoglutarate promoted wheat growth and dry matter accumulation under drought stress^25^. Similarly, our data indicated that exogenous application of α-oxoglutarate has positive effect on fresh weight of soybean seedling under cold stress.

**Table 1:** Effects of α-oxoglutarate on fresh weight of soybean (g plant^-1^).

Treatment	S1 stage		S2 stage	
	Henong60	Heinong48	Henong60	Heinong48
A_0_	1.825	2.209	1.709	1.903
A_2.5_	1.835	2.265	1.724	1.938
A_5.0_	1.861	2.294	1.772	1.959
A_7.5_	1.842	2.212	1.741	1.964
ANOVA effect				
A	ns	ns	ns	ns
V	***		**	
A × V	ns		ns	

Means with same letter are not significantly different (*P* < 0.05) using LSD Test. ***, **, *, and ns denote significance at 0.001, 0.01, 0.05 and not significant (*P* > 0.05), respectively.

### Response of leaf proline content to exogenous α-oxoglutarate

As shown in Table 2, significant impact of exogenous α-oxoglutarate on leaf proline content was observed at S1 and S2 stages. There was a significant difference in proline content between two varieties at both stages. The leaf proline content for Henong60 was significantly higher than that for Heinong48 under cold stress at S1 and S2 stages. Leaf proline content at A_2.5_, A_5.0_, and A_7.5_ was significantly greater than control at both stages. Leaf proline content first increased and then decreased with increasing exogenous α-oxoglutarate concentration and A_5.0_ produced the highest proline content for two soybean varieties at S1 and S2 stages. Leaf proline content for Henong60 and Heinong48 at A_5.0_ was 37.53% and 17.96% higher than that at A_0_ at S1 stage, respectively. Leaf proline content for Henong60 and Heinong48 increased by 28.82 and 12.41% at A_5.0_ and A_0_, respectively, at S2 stage.

**Table 2:** Effects of α-oxoglutarate on leaf proline content (µg g^-1^ FW).

Treatment	S1 stage		S2 stage	
	Henong60	Heinong48	Henong60	Heinong48
A_0_	36.26c	31.01b	35.65c	27.65b
A_2.5_	45.42b	36.12a	43.58b	30.37a
A_5.0_	49.87a	36.58a	45.21a	31.08a
A_7.5_	45.31b	35.82a	43.85b	30.85a
ANOVA effect				
A	***	*	***	**
V	***		***	
A × V	**		**	

Proline accumulation, a common physiological response of plants exposed to cold stress, is considered to play adaptive roles in stress tolerance^26^. The role of proline in cold tolerance has been investigated for decades and positive correlations between proline accumulation and improved cold tolerance have been reported^27, 28^. Proline has been proposed to act as a compatible osmolyte and to be a way to store carbon and nitrogen^29^. Finally proline accumulation may be part of the stress signal influencing adaptive responses^30^. Our results indicated that leaf proline content for cold-resistant variety Henong60 was significantly higher than that for cold-sensitive variety Heinong48 (Table 2). It is important to take preventive actions to alleviate the adverse effect of abiotic stress. Our results indicated that exogenous α-oxoglutarate significantly increased proline content at S1 and S2 stages (Table 2). An early published report by Li et al.^20^ stated that exogenous application of α-oxoglutarate could alleviate the adverse effect of drought stress on wheat growth and increase the proline content in wheat leaf. Their results disclosed the importance of exogenous α-oxoglutarate under abiotic stress.

### Response of leaf glutamate content to exogenous α-oxoglutarate

It is important to note that glutamate content in leaf was significantly influenced by α-oxoglutarate, variety and their interaction (Table 3). The leaf glutamate content was significantly greater for Henong60 than for Heinong48 under cold stress at S1 and S2 stages. Leaf glutamate content at A_2.5_, A_5.0_, and A_7.5_ was significantly greater than that at A_0_ at S1 and S2 stages. There was no significant difference in glutamate content for Heinong48 among A_2.5_, A_5.0_, and A_7.5._ Leaf glutamate content first increased and then decreased with the decrease of exogenous α-oxoglutarate concentration and A_5.0_ gave the highest glutamate content for two soybean varieties at S1 and S2 stages. Leaf glutamate content for Henong60 and Heinong48 increased by 39.59% and 21.32% at A_5.0_ than at A_0_ at S1 stage, respectively. Leaf glutamate content for Henong60 and Heinong48 at A_5.0_ was 32.68% and 22.13% higher than that at A_0_ at S2 stage, respectively.

**Table 3:** Effects of α-oxoglutarate on leaf glutamate content (µg g^-1^ FW).

Treatment	S1 stage		S2 stage	
	Henong60	Heinong48	Henong60	Heinong48
A_0_	132.12c	89.16b	122.86c	69.01b
A_2.5_	150.44b	105.96a	140.98b	83.17a
A_5.0_	184.43a	108.17a	163.01a	84.28a
A_7.5_	155.58b	103.32a	142.85b	82.92a
ANOVA effect				
A	***	***	***	***
V	***		***	
A × V	**		*	

Glutamate is the major amino acid used for proline biosynthesis through a constant glutamate pool^31^. A substantial supply of glutamate is needed when the rate of proline biosynthesis is increased^26^. Supplying α-ketoglutarate for plant has a positive effect on the increase of glutamate content^6^ and in our study (Table 3).

### Response of leaf GS and GDH activity to exogenous α-oxoglutarate

The data obtained in this study showed that the response of leaf GS activity to exogenous α-oxoglutarate was different under cold stress (Table 4). Exogenous application of α-oxoglutarate posed significant impact on GS activity in leaf for two soybean varieties at S1 and S2 stages. There was a significant difference in GS activity between two varieties at each stage. The leaf GS activity for Henong60 was significantly higher than that for Heinong48 under cold stress at S1 and S2 stages. Leaf GS activity at A_2.5_, A_5.0_, and A_7.5_ was significantly greater than that at A_0_ at S1 and S2 stages. Leaf GS activity first increased and then decreased with a decrease in α-oxoglutarate concentration and A_5.0_ gave the highest GS activity for two soybean varieties at S1 and S2 stages. Leaf GS activity at A_5.0_ than for Henong60 and Heinong48 increased by 40.54% and 27.21% at A_5.0_ than at A_0_ at S1 stage, respectively. Leaf GS activity for Henong60 and Heinong48 under A_5.0_ was 68.01% and 56.05% higher than that under A_0_ at S2 stage, respectively.

**Table 4:** Effects of α-oxoglutarate on leaf GS activity (µmol h^-1^ g^-1^ FW).

Treatment	S1 stage		S2 stage	
	Henong60	Heinong48	Henong60	Heinong48
A_0_	202.32c	179.56c	140.98c	120.01b
A_2.5_	251.12b	215.68ab	210.85b	180.17a
A_5.0_	284.35a	228.42a	236.86a	187.28a
A_7.5_	265.50b	212.33b	209.01b	184.92a
ANOVA effect				
A	***	***	***	***
V	***		***	
A × V	**		*	

Results from this study demonstrated that leaf GDH activity was significantly affected by exogenous α-oxoglutarate, variety and their interaction at S1 and S2 stages (Table 5). There was a significant difference in GDH activity between two varieties at each stage. The leaf GDH activity first increased and then decreased with an increase in exogenous α-oxoglutarate concentration and A_5.0_ gave the highest GDH activity for two soybean varieties at S1 and S2 stages. Leaf GDH activity for soybean varieties decreased with time after exposure to exogenous α-oxoglutarate. From S1 stage to S2 stage, leaf GDH activity for cold-resistant variety Henong60 and cold-sensitive variety Heinong48 decreased by 18.46% and 20.96%, respectively. Results in this study clearly indicated that there was lower reduction in GDH activity for cold-resistant variety Henong60 than cold-sensitive variety Heinong48 under cold stress.

**Table 5:** Effects of cold stress on leaf NADH-GDH activity (µmol h^−1^ g^−1^ FW).

Treatment	S1 stage		S2 stage	
	Henong60	Heinong48	Henong60	Heinong48
A_0_	13.26c	11.12b	11.53c	8.51b
A_2.5_	16.55b	13.58a	14.32b	11.68a
A_5.0_	19.66a	14.01a	15.21a	12.01a
A_7.5_	16.57b	13.86a	14.69b	11.26a
ANOVA effect				
A	***	***	***	***
V	***		***	
A × V	*		**	

Previous studies have indicated that the GS/GOGAT cycle plays a major role in glutamate production when proline is needed^32^. In many studies, enhanced NADH-GDH activity and its important role in supplying glutamate for proline synthesis have been revealed^33, 34^. Oliveira et al.^35^ reported that exogenous application of α-ketoglutarate modulated the expression of glutamine synthetase in *Arabidopsis*. Under drought stress, foliar application of α-ketoglutarate increased the activity of GS and GDH in wheat leaf^17^. The data in our study clearly demonstrated that α-ketoglutarate had positive effect on the increase of GS and GDH activity for soybean seedling exposed to cold stress (Tables 4 and 5). Increased activity of GS and GDH caused by exogenous explain the reason why ammonium content in soybean leaf is accordlingly reduced when soybean seedling is exposed to cold stress in our study (Tables 4, 5, 6). The effect of α-ketoglutarate on nitrogen assimilation was also reflected by a markedly lower level of ammonium in our study.

**Table 6:** Effects of cold stress on leaf ammonium content (µmol g^−1^ FW).

Treatment	S1 stage		S2 stage	
	Henong60	Heinong48	Henong60	Heinong48
A_0_	1.41a	1.76a	1.71a	2.33a
A_2.5_	1.16b	1.44b	1.34b	1.79b
A_5.0_	0.91c	1.38b	1.12c	1.81b
A_7.5_	1.18b	1.42b	1.38b	1.85b
ANOVA effect				
A	***	**	***	***
V	**		***	
A × V	*		*	

### Response of leaf ammonium content to exogenous α-oxoglutarate

Excess ammonium can lead to toxic effect on plant. As shown in Table 6, leaf ammonium content was significantly decreased by exogenous application of α-oxoglutarate. Leaf ammonium content for both varieties showed a lower increase with time after exposure to exogenous α-oxoglutarate than control. Leaf ammonium content for Henong60 was lower than that for Heinong48 at each stage. The leaf ammonium content first decreased and then increased with an increase in exogenous α-oxoglutarate concentration and A_5.0_ gave the lowest ammonium content for two soybean varieties at S1 and S2 stages.

Excessive ammonium is toxic to plants and must be rapidly transformed into non-toxic organic nitrogen compounds. It has been demonstrated that GS/GOGAT cycle is the major route of ammonium assimilation in plants. Alternatively, NADH-dependent glutamate dehydrogenase (NADH-GDH) can also catalyze ammonium incorporation into glutamate^7, 8^. The function of the alternative GDH pathway is believed to play a complementary role under conditions of environmental stress or excessive ammonia supply^10, 11^. Our data indicated that foliar application of α-oxoglutarate increased GS activity, GDH activity, assimilated more ammonium and led to decreased ammonium content (Tables 4, 5, 6). Similarly, Magalhaes et al.^21^ reported that NH_4_^+^ concentrations in tomato roots and shoots with exogenous α-oxoglutarate sharply decreased compared to control and the increase of NH_4_^+^ assimilation due to exogenous α-oxoglutarate clearly indicated that the availability of carbon skeletons is a key limiting factor for NH_4_^+^ assimilation in tomato plants. Yuan et al.^17^ investigated the role of α-oxoglutarate in regulation of carbon and nitrogen metabolisms in rice exposed to abiotic stress and found that exogenous α-oxoglutarate enhanced GS activity. And the increase of ammonium uptake was also observed in their study^17^. An early report of Brugiere et al.^36^ confirmed that proline synthesis might be a means of assimilating excessive ammonium. Our study indicated that foliar application of α-oxoglutarate significantly increased proline content and decreased ammonium content (Tables 2, 6).

2-oxoglutarate (2-OG) is a key metabolite at the crossroads of carbon/nitrogen metabolism as it is required for ammonia assimilation^13–15^. The physiological mechanism by which α-oxoglutarate alleviates the adverse effect of cold on soybean seedlings in the present study is that foliar application of α-oxoglutarate increased GS activity, GDH activity, assimilated more ammonium and led to decreased ammonium content and synthesized more glutamate which is necessary for proline biosynthesis. In the end, an increase in proline content could explain the reason why α-oxoglutarate alleviates the adverse effect of cold on soybean seedlings.

### Response of chlorophyll content and photosynthetic rate to exogenous α-oxoglutarate

Results from this study indicated that leaf chlorophyll content was significantly affected by exogenous α-oxoglutarate, variety and their interaction at S1 stage and S2 stage under cold stress (Table 7). The leaf chlorophyll content was significantly higher for Henong60 than Heinong48 under cold stress at both stages. The leaf chlorophyll content at A_2.5_, A_5.0_, and A_7.5_ was significantly higher than that at A_0_ at S1 and S2 stages. In the present study, A_5.0_ gave the highest chlorophyll content for two soybean varieties at S1 and S2 stages. There was no significant difference in chlorophyll content for two soybean varieties between A_2.5_ and A_7.5_ at S1 stage.

**Table 7:** Effects of α-oxoglutarate on leaf chlorophyll content (*SPAD*).

Treatment	S1 stage		S2 stage	
	Henong60	Heinong48	Henong60	Heinong48
A_0_	41.32c	39.08c	38.86c	35.23b
A_2.5_	49.60b	41.17b	47.29b	37.21a
A_5.0_	52.18a	43.95a	49.79a	37.68a
A_7.5_	49.92b	41.08b	46.83b	37.02a
ANOVA effect				
A	***	***	***	**
V	***		**	
A × V	**		**	

The photosynthetic rate is an important parameter of photosynthesis. The data in Table 8 demonstrated that leaf photosynthetic rate was significantly influenced by exogenous α-oxoglutarate, variety and their interaction at S1 and S2 stages under cold stress. The leaf photosynthetic rate was significantly greater for Henong60 than Heinong48 under cold stress at each stage. The leaf photosynthetic rate was significantly greater at A_2.5_, A_5.0_, and A_7.5_ than at A_0_ at both S1 and S2 stages. In our study, A_5.0_ produced the highest photosynthetic rate for two soybean cultivars at S1 and S2 stages. There was no significant difference in photosynthetic rate for two soybean varieties between A_2.5_ and A_7.5_ at S1 stage.

**Table 8:** Effects of α-oxoglutarate on photosynthetic rate (µmol CO_2_ m^−2^ s^−1^).

Treatment	S1 stage		S2 stage	
	Henong60	Heinong48	Henong60	Heinong48
A_0_	21.36c	20.08c	19.65c	17.32b
A_2.5_	24.52b	21.17b	23.32b	18.29a
A_5.0_	26.13a	23.95a	24.69a	18.65a
A_7.5_	24.81b	21.08b	23.87b	18.04a
ANOVA effect				
A	***	***	***	**
V	**		***	
A × V	**		**	

α-ketoglutarate plays an important role in nitrogen and carbon metabolism in plants^13^. The chlorophyll content and photosynthetic rate are important parameters of photosynthesis. Many studies indicated that exogenous application of α-ketoglutarate had positive effect on photosynthesis^36–38^. Our results supported the work conducted by Ge^37^ who reported that α-oxoglutarate significantly increased leaf chlorophyll content and photosynthetic rate in winter-wheat seedling exposed to drought stress. Foliar application of α-oxoglutarate increased GS activity, GDH activity, assimilated more ammonium and led to increase in proline content for both varieties at S1 and S2 stages. Because there is a great need of NADPH when proline is synthesized, NADP^+^ will be produced^39^. As known to all, NADP^+^ is necessary to photosynthesis. Therefore, we infer that exogenous α-oxoglutarate increased photosynthetic rate by enhancing proline content.

## Conclusions

Exogenous α-oxoglutarate significantly increased leaf GS activity, NADP-GDH activity, glutamate content, proline content and photosynthesis of soybean seedling exposed to cold stress at S1 and S2 stages. Accordlingly, the ammonium content was significantly decreased by exogenous α-oxoglutarate at S1 and S2 stages. 5.0 mmol/L of exogenous α-oxoglutarate is the optimum concentration in our study. There was a significant difference in leaf GS activity, NADP-GDH activity, glutamate content, proline content and photosynthesis between Henong60 and Heinong48 under cold stress at S1 and S2 stages. Those results suggested that exogenous α-oxoglutarate could alleviate the adverse impact of cold stress. In our region, cold stress usually not existed more than two days. Therefore, it is noted that foliar application of α-oxoglutarate is a feasible way to prevent soybean against cold stress in Northeast China.
